# EXercising with Computers in Later Life (EXCELL) - pilot and feasibility study of the acceptability of the Nintendo^® ^WiiFit in community-dwelling fallers

**DOI:** 10.1186/1756-0500-3-238

**Published:** 2010-09-13

**Authors:** Marie A Williams, Roy L Soiza, Alison McE Jenkinson, Alison Stewart

**Affiliations:** 1 Department of Medicine for the Elderly, NHS Grampian, DOME Office, Woodend Hospital, Eday Road, Aberdeen, AB15 6XS, Scotland, UK; 2 School of Medical Sciences, University of Aberdeen, IMS Building, Foresterhill, Aberdeen, AB25 2ZD, Scotland, UK; 3 Bone and Musculoskeletal Research Programme, School of Medicine, University of Aberdeen, Foresterhill, Aberdeen, AB25 2ZD, Scotland, UK

## Abstract

**Background:**

Falls management programmes have been instituted to attempt to reduce falls. This pilot study was undertaken to determine whether the Nintendo^® ^WiiFit was a feasible and acceptable intervention in community-dwelling older fallers.

**Findings:**

Community-dwelling fallers over 70 years were recruited and attended for computer-based exercises (n = 15) or standard care (n = 6). Balance and fear of falling were assessed at weeks 0, 4 and 12. Participants were interviewed on completion of the study to determine whether the intervention was acceptable.

Eighty percent of participants attended 75% or more of the exercise sessions. An improvement in Berg Score was seen at four weeks (p = 0.02) and in Wii Age at 12 weeks (p = 0.03) in the intervention group. There was no improvement in balance scores in the standard care group.

**Conclusion:**

WiiFit exercise is acceptable in self-referred older people with a history of falls. The WiiFit has the potential to improve balance but further work is required.

**Trial Registration:**

ClinicalTrials.gov - NCT01082042

## Background

Community dwelling individuals, over the age of 65, fall at a rate of 35% per year [1], and this increases with advancing age [2]. Falls have significant implications for both the faller and the population, including the development of "fear of falling", which can have a significant impact on individuals with self-imposed decline in function and activity avoidance [3].

UK/US falls guidelines support referral of fallers for multidisciplinary assessment [1,4,5]. Significant improvements in balance have been noted in trials of exercise interventions involving gait, balance, coordination, functional exercises and muscle strengthening [6]. However, the optimal mode, duration and intensity of exercise to reduce falls remain uncertain.

The Nintendo^® ^WiiFit ("WiiFit") is a software/hardware game package for the Nintendo^® ^Wii, designed to improve balance and fitness, whilst providing entertainment (further information in appendix 1). There is a lack of evidence showing the acceptability of interactive computer games in the older faller.

This purpose of this pilot study was to determine the feasibility and acceptability of the WiiFit in community-dwelling older fallers and whether it has the potential to improve balance. Given the lack of published evidence it would be unethical to withhold standard care in a randomised controlled trial.

## Methods

This longitudinal intervention study was undertaken in community dwelling people over the age of 70 years who had fallen in the previous year. We compared 2 groups: an intervention group who attended for WiiFit exercise sessions and a standard care group who attended the local falls group.

### Inclusion and Exclusion Criteria

#### Inclusion criteria

Age 70 years or older, living locally in the community (in sheltered accommodation or own home), fallen at least once in the preceding 12 months and have an abbreviated mental test (AMT) [7] of seven or over (maximum score 10).

#### Exclusion criteria

Wheelchair bound individuals, people living in a care homes or long-term hospital care and current enrolment in an exercise or rehabilitation programme.

### Patient Recruitment

#### Intervention group

A press release was issued to recruit members to the intervention group. This resulted in widespread press coverage. Fifty-three potential participants contacted the research team, of these 14 were excluded as they had not fallen in the preceding 12 months. The first 15 eligible people were included in the study.

#### Standard Care

People referred to the local falls group were approached by the research team. Of 13 potential recruits, one was excluded as a result of low AMT, two refused to participate in standard care and four declined to participate in the study. Thus six participants were recruited. Due to poor uptake/high drop-out rates recruitment was abandoned after three months.

### Ethical Approval and Trial Registration

The North of Scotland Research Ethics Committee granted ethical approval (Ref No. 08/S0801/186). The study was registered with ClinicalTrials.gov (NCT01082042).

### Assessment Measures

At an initial interview baseline information was obtained. Balance was assessed by a physiotherapist at weeks 0, 4 and 12. Functional balance was assessed by the Berg Balance Scale (BBS), a performance based measure using 14 activities of daily living (range 0-56) [8]. A Tinetti Balance Assessment Tool (part of the Performance Orientated Mobility Assessment) was also undertaken (range 0-16) [9]. Higher scores on both scales indicate better balance and both appear to have good inter- and intra- rater reliability [8,9].

The Falls Efficacy Scale - International (FES-I), which is reliable and valid [10], was undertaken at weeks 0, 4 and 12 to assess the degree of concern about falling in certain situations. Higher scores indicate more concern about an individual falls risk (range 16-64).

Acceptability of the intervention was assessed by monitoring attendance, completion of an Attitude to Falls-Related Interventions Scale (AFRIS) and a qualitative interview. The AFRIS is a validated measure of the acceptability of falls-related interventions (range 6-42, higher scores reflect a greater degree of positivity about the intervention) [11]. All members of the intervention group were interviewed on completion or withdrawal from the study to determine acceptability of the WiiFit (appendix 2). The interview was undertaken, and transcribed, by one of the researchers. Two independent raters undertook thematic analysis of these interviews.

#### Intervention group only

The intervention group had their WiiFit Age calculated by the WiiFit software (based on the user's current age, weight and athletic ability) undertaken at weeks 0 and 12 [12].

### The Exercise Programme

#### Intervention group

The intervention group attended individual exercise visits supervised by a member of the research team twice weekly for 12 weeks. A walking frame was placed in front of the balance board, if the participant deemed this necessary. The exercise programme included balance and aerobic exercises on the WiiFit (appendix 1) and was modified at weeks 4 and 8. Participants were allowed to stop and move onto the next exercise at any point (including if they could not manage a particular exercise) and were allowed to terminate the session at any point. They were advised not to play the Wii out with the study. The actual time exercised was measured for each session.

#### Standard Care group

The standard care group was recruited from the local falls group, which is run by the local NHS Hospital. Patients attend a 12-week exercise/education programme supervised by NHS physiotherapists.

### Statistics

SPSSv16.0 (Chicago, IL) was used to analyse the data. Normally distributed data were compared using Independent samples T-Test between groups and equal variances were assumed. AMT scores were compared using Mann-Whitney U-Test. Fisher's exact test was used to compare living situation and use of walking aid, and Pearson-Chi Squared test was used to compare number of falls. Paired samples T-Test was used to assess change in the formal balance scores following the exercise programmes.

## Results

### Baseline Characteristics

**Figure 1:**
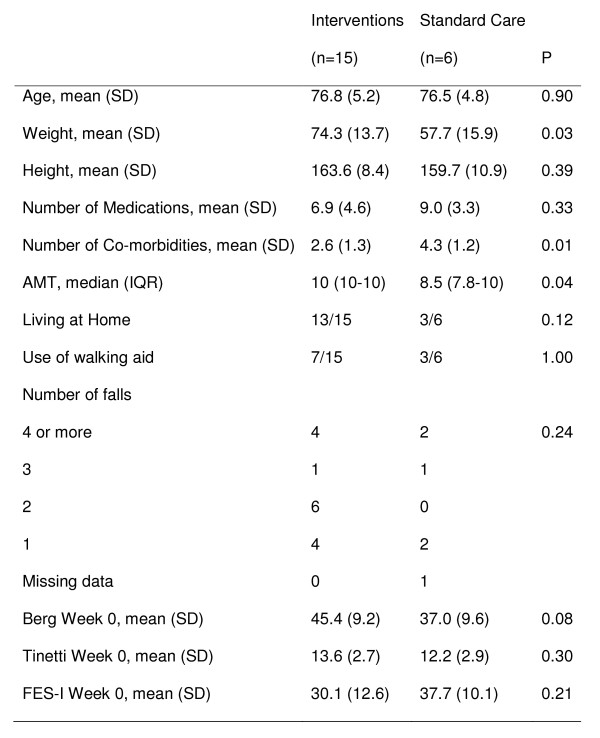
Baseline Characteristics

Figure 1 provides the baseline characteristics of both intervention and standard care groups. The standard care group were found to have statistically significant lower weight, higher number of co-morbidities and lower AMT score.

One member of the intervention group had previous experience of the Wii but had not played the WiiFit. Two of the intervention group had previously attended a falls group (equivalent to standard care).

### Acceptability

**Figure 2:**
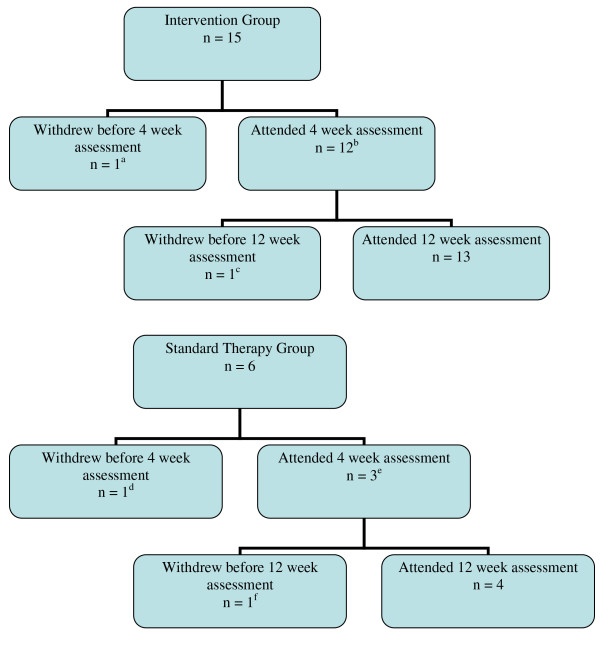
**Follow up of trial participants **a = due to knee pain b = 2 participants did not attend for 4 week assessment but attended subsequent visits c = due to death of spouse (NB this participant did not attend for 4 week assessment) d = admitted to hospital e = 2 participants did not attend for 4 week assessment but attended subsequent visits f = admitted to hospital

On study completion there were 13 (86.7%) in the intervention group and 4 (66.7%) in standard care. Follow up is shown in figure 2.

The mean number of sessions completed by the intervention group was 19 (range=3 - 24, maximum 24). Attendance was good with 80% of participants attending 75% or more of the sessions. No significant difference in AFRIS score (p = 0.45) between the intervention group (Mean (SD) = 34.3 (7.2)) and the standard care group (Mean (SD) = 37.3 (3.3)) were found.

At interview we found that 100% of the intervention group (inclusive of the two dropouts) found the intervention to be enjoyable and acceptable. Over half of participants felt that the exercise sessions were the right length and the right frequency (69%), with 23% expressing that the sessions were too short. The intervention group felt that "people like me" would participate in a similar exercise programme if it was more widely available (77%). Participants expressed a strong desire to exercise with the WiiFit in the future (92%), showing no preference towards exercising on their own/with company or towards supervised/unsupervised sessions. When asked for a preference to falls group or exercise with the WiiFit in the future, 61% chose the WiiFit, 8% chose falls group, 8% chose both and 23% did not know.

A number of recurring themes were identified during the interviews. Positive themes were an improvement in confidence following the intervention, a sense of positivity about the researchers and the company the sessions provided and that the participants would like a Wii of their own. Negative themes identified were pain or discomfort, feeling tired and difficulties getting to the sessions.

There was no difference between FES-I at the start and end of the study in the intervention group. However, there was a trend towards increasing FES-1 scores in the standard care group on completion of the study.

### Study Feasibility

For a future trial to be feasible the target population must be able to comply with the intervention. A low number of adverse events were recorded. One participant fell backwards (no injuries sustained) when stepping from the balance board, and another felt temporarily lightheaded on completion of one of the exercise sessions.

**Figure 3:**
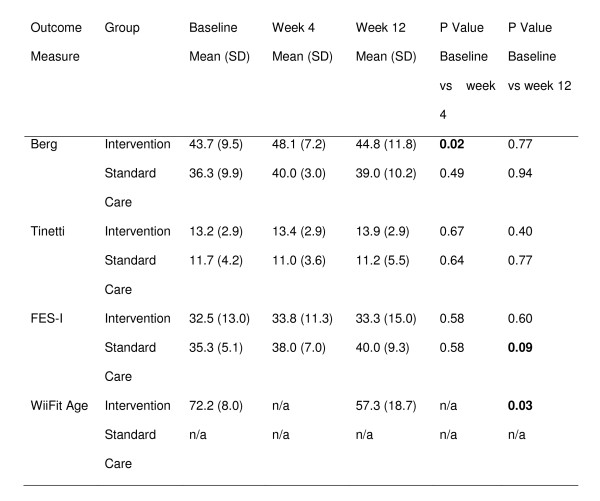
Berg, Tinetti and FES-I scores at baseline, 4 weeks and 12 weeks

We sought to determine whether the WiiFit has the potential to improve balance and whether this is comparable to standard care. The results are shown in figure 3. A statistically significant improvement in BBS was seen at four weeks (p = 0.02) and in Wii Age at 12 weeks (p = 0.03) in the intervention group. Six intervention (40%) and 5 standard care (83.3%) participants had a BBS of less than 45. Thirty-three percent of participants who completed a 4 week assessment had a change in BBS of 8 or more.

## Discussion

This pilot study is one of the first to look at acceptability of the WiiFit as a falls intervention in older adults. There are low levels of willingness of older adults to participate in group strength and balance training [13]. In our study, there was no difference in AFRIS score suggesting that the WiiFit is as acceptable as standard care. For future trials to be feasible the target population must be able to comply with the intervention. This pilot study has shown that older community-dwelling fallers are willing to attend exercise sessions with the WiiFit as shown by the high attendance, low drop-out rate and positive responses obtained in the qualitative interviews. In addition, a low number of adverse events occurred during the exercise sessions.

A randomised controlled trial would probably be feasible given our findings, and is essential to establish whether exercise with the WiiFit is, at least, as beneficial as standard care. As the standard care group represented a frailer population, we were unable to effectively compare standard care and the WiiFit intervention. However, the potential to improve balance in this specified population was seen, as there was a significant improvement in BBS at 4 weeks in the intervention group. Conradsson *et al *have suggested that a change of 7.7 BBS score points is required to reveal a genuine modification of function between 2 assessments [14]. In both standard therapy and the intervention group 33% of participants who completed a 4 week assessment had a change in BBS of 8 or more. Thus it is plausible that both intervention and standard therapy have the potential to improve BBS. Improvement in intervention group BBS was not sustained at 12 weeks. It is possible that this phenomenon is a reflection of the lack of the study's statistical power. A further possibility is that there was an inadequate increase in exercise intensity over the study period. The games used in this study were selected on the basis that they were 'open' to all users from the initial session and the researchers felt they would be acceptable to the study participants. However, a number of participants expressed that a wider range of games might have prevented boredom. The reason for the lack of sustained improvement in BBS remains unclear. In the intervention group a statistically significant decrease in WiiFit age was found - also suggesting an improvement in balance. However WiiFit age is not a validated measure and further work would be required to valid this finding.

There was no change in FES-I in the intervention group at 4 weeks despite improvement in BBS. Furthermore a significant deterioration in FES-I score was seen in the standard care group at 12 weeks. The lack of improvement in balance confidence despite improvement in balance ability has been previously shown [15]. It is plausible that as a result of attending a falls intervention programme, perception of falls risk is heightened and this is reflected in a higher FES-I score. Falls and confidence measures are subjective and thus the participants score will be dependent on how individuals feel at time of completion of the score only. Interestingly our qualitative data for the intervention group suggested that the participants' confidence had improved. It is not clear why this discrepancy between qualitative data and FES-I score has occurred.

Further work must be undertaken to determine whether exercise with the WiiFit should be included in falls prevention programmes. If improvements in balance can be shown then the WiiFit could be used as part of supervised physiotherapy based exercise sessions or within the patients' own homes. With advances in telemedicine it is plausible that participants may be able to undertake exercise sessions within their own homes with the therapists monitoring progress from their base hospital. This may prove more cost-effective than standard care due to reductions in transport and staffing costs.

### Study Limitations

The standard therapy group are likely to represent a frailer population in that there are statistically significant differences in weight, number of co-morbidities and AMT score, and a trend towards difference in baseline BBS. Difficulties in recruitment and a high drop out rate in the standard care group led to problems with comparison of the groups.

A further limitation is that our intervention group had a mean BBS at baseline of 45 and Berg *et al *have previously determined that a BBS of 45 or more indicates that an individual is likely to be safe in independent ambulation [8]. Thus any target population for falls interventions should have a Berg score at baseline of 45 or less.

In addition WiiFit sessions were undertaken in a supervised hospital environment and are unlikely to be reflective of people exercising with the WiiFit in their own homes. However participants indicated they were willing to exercise with the Wii in their own homes. This is important as research has found that older people are more open to exercise programmes undertaken in their own home [13].

## Conclusions

The WiiFit appears to be an acceptable falls intervention in the community dwelling older individuals who have fallen and has the potential to improve balance and self-perceived confidence. Future work must be undertaken to determine an acceptable exercise programme with the greatest potential to improve balance. A randomised controlled trial is required to determine whether the WiiFit is comparable to standard care.

## Competing interests

The authors declare that they have no competing interests.

## Authors' contributions

MAW contributed to the data collection, coordinated the study, contributed to the statistical analysis and led the writing of the manuscript. RLS participated in the design of the study, and contributed to the statistical analysis and manuscript writing. AMcEJ contributed to the design of the study and manuscript writing. AS conceived the study, participated in its design and contributed to the statistical analysis and manuscript writing. All authors read and approved the final manuscript.

## Appendix 1

### The Nintendo^® ^Wii and Nintendo^® ^WiiFit

The Wii is a video game console which detects movement allowing the individual to play interactive games. WiiFit is an exercise game which combines a balance board on which the individual stands (a flat board that looks like bathroom weight scales) with the Wii games system. The balance board incorporates pressure sensors to monitor centre of balance and shifts in weight/balance.

See the following for further information: http://www.nintendo.com/wii

http://wiifit.com

http://en.wikipedia.org/wiki/Wii

http://en.wikipedia.org/wiki/Wii_Fit

### Exercise Programme Week 0 - 4

1. Aerobic games - Jogging - 1 attempt to half way on the circuit

2. Balance games - Tilt Table - 3 attempts

3. Aerobic games - Step Basics - 2 attempts up to the point where the feet turn blue

4. Balance games - Ski Slalom - 5 attempts

5. Game of own choice from list above - 1 attempt

6. Yoga game - Breathing Exercise - 1 attempt

All games played at beginner level.

### Exercise Programme Week 4 - 8

1. Aerobic games - Jogging - 1 attempt to half way on the circuit

2. Balance games - Tilt Table - 2 attempts - option of advanced level

3. Aerobic games - Step Basics - 2 attempts up to the point where the feet turn blue

4. Balance games - Heading - 2 attempts - beginner level

5. Balance games - Ski Slalom - 5 attempts - option of advanced level

6. Balance games - Ski Jump - 2 attempts - beginner level

7. Yoga game - Breathing Exercise - 1 attempt

### Exercise Programme Week 8 - 12

1. Aerobic games - Jogging - 1 attempt to half way on the circuit

2. Balance games - Tilt Table - 2 attempts - option of advanced level

3. Aerobic games - Step Basics - 2 attempts up to the point where the feet turn blue

4. Balance games - Heading - 2 attempts - option of advanced level

5. Aerobic games - Hula Hoop - 1 attempt - beginner level

6. Balance games - Ski Slalom - 3 attempts - option of advanced level

7. Balance games - Ski Jump - 2 attempts - option of advanced level

8. Yoga game - Breathing Exercise - 1 attempt

## Appendix 2

### Interview Schedule for Interventions who Completed Study

1. Do you feel your balance has improved following the balance training with the Wii?

2. How did you feel about the exercise sessions?

3. Was the Wii an acceptable form of exercise to you?

4. What did you think about the length of the exercise sessions?

5. What did you think about the frequency of the exercise sessions?

6. Do you think people like you would participate in an exercise programme like this if it was more widely available

7. Would you undertake exercise with the Wii again?

a. Yes or No?

b. On your own or with others?

c. Supervised or unsupervised?

8. Anything else you would like to say about the exercise sessions?

9. If given the option of falls group or Wii in the future what would you chose?

10. Any falls or problems during the study

### Interview Schedule for Interventions who Withdrew from Study

1. Why did you withdraw from the study?

2. Do you feel your balance has improved following the balance training with the Wii?

3. How did you feel about the exercise sessions?

4. Was the Wii an acceptable form of exercise to you?

5. What did you think about the length of the exercise sessions?

6. What did you think about the frequency of the exercise sessions?

7. Do you think people like you would participate in an exercise programme like this if it was more widely available?

8. Would you undertake exercise with the Wii again?

a. Yes or No?

b. On your own or with others?

c. Supervised or unsupervised?

9. Anything else you would like to say about the exercise sessions?

10. If given the option of falls group or Wii in the future what would you chose?
